# Shaping Sustainable Urban Environments by Addressing the Hydro-Meteorological Factors in Landslide Occurrence: Ciuperca Hill (Oradea, Romania)

**DOI:** 10.3390/ijerph18095022

**Published:** 2021-05-10

**Authors:** Cezar Morar, Tin Lukić, Biljana Basarin, Aleksandar Valjarević, Miroslav Vujičić, Lyudmila Niemets, Ievgeniia Telebienieva, Lajos Boros, Gyula Nagy

**Affiliations:** 1 Department of Geography, Tourism and Territorial Planning, University of Oradea, 410087 Oradea, Romania; 2 Department of Geography, Tourism and Hotel Management, University of Novi Sad, 21000 Novi Sad, Serbia; lukic021@gmail.com (T.L.); biljana.basarin@gmail.com (B.B.); vujicicm@gmail.com (M.V.); 3 Faculty of Geography, University of Belgrade, 11000 Belgrade, Serbia; aleksandar.valjarevic@gef.bg.ac.rs; 4 Department of Human Geography and Regional Studies, V.N. Karazin Kharkiv National University, 61022 Kharkiv, Ukraine; ludmila.nemets@karazin.ua (L.N.); eugene.telebeneva@karazin.ua (I.T.); 5 Department of Economic and Social Geography, University of Szeged, 6720 Szeged, Hungary; borosl@geo.u-szeged.hu (L.B.); geo.nagy.gyula@gmail.com (G.N.)

**Keywords:** geohazard, landslide, slope instability, rainfall erosivity, environmental sustainability, Oradea, Romania

## Abstract

Romania is one of the countries severely affected by numerous natural hazards, where landslides constitute a very common geomorphic hazard with strong economic and social impacts. The analyzed area, known as the “Ciuperca Hill”, is located in Oradea (NW part of Romania) and it has experienced a number of landsliding events in previous years, which have endangered anthropogenic systems. Our investigation, focused on the main causal factors, determined that landslide events have rather complex components, reflected in the joint climatological characteristics, properties of the geological substrate, and human activity that further contributed to the intensive change of landscape and acceleration of slope instability. Analysis of daily precipitation displays the occurrence and intensive distribution between May and September. Higher values of rainfall erosivity (observed for the 2014–2017 period), are occurring between April and August. Erosivity density follows this pattern and indicates high intensity events from April until October. *SPI* index reveals the greater presence of various wet classes during the investigated period. Geological substrate has been found to be highly susceptible to erosion and landsliding when climatological conditions are suitable. Accelerated urbanization and reduced vegetation cover intensified slope instability. The authors implemented adequate remote-sensing techniques in order to monitor and assess the temporal changes in landslide events at local level. Potential solutions for preventative actions are given in order to introduce and conduct qualitative mitigation strategies for shaping sustainable urban environments. Results from this study could have implications for mitigation strategies at national, regional, county, and municipality levels, providing knowledge for the enhancement of geohazard prevention and appropriate response plans.

## 1. Introduction

### 1.1. Urban Areas and Sustainability

Europe is a network of a majority of highly populated urban areas [1]. Cities have surpassed their traditional role as economic engines of regional growth; they currently strive to provide amenities such as cultural, educational or health services, all part of the modern urban lifestyle, which includes sustainability, well-being, and inclusive growth. In meeting their needs for food, energy, space and other resources, these densely urbanized areas are subject to significant pressures and challenges [2] like urban sprawl, scattered development, urban dispersion, or soil sealing [3,4]. All these are endangering the process of sustainable urban development, affecting local socio-economic and environmental systems. In Europe, the urban population is expected to rise to 80% by 2050 [5], adding additional pressures on urban systems. The concentration of economic activities and people in cities significantly affects the environment, challenging sustainability and undermining resilience [6]. In this context, natural capital is under tremendous pressure, influencing the overall quality of urban life, directly influenced by the quality of the environment [7]. In the same perspective, putting back into productive operation the under-used, degraded, or abandoned land becomes more important than ever. To meet sustainability goals cities have to be planned and managed in an increasingly efficient way [8,9]. Therefore, developing and supporting natural solutions, or Green Infrastructure, bring a variety of environmental, economic and social benefits, versus the more expensive to operate and maintain “grey infrastructure”. Green Infrastructure (GI) is based on the principle of “protecting and enhancing nature and natural processes (…) are consciously integrated into spatial planning and territorial development” [10]. Green Infrastructure relies on economically based construction solutions, as nature provides much cheaper and sustainable solutions. Integrating nature-based solutions in the local urban settings is a cost-effective process that preserves the physical features of the environment and enhances the local identity [11]. In addition, the Green Infrastructure’s beneficial solutions [12] provide health-related benefits (clean air and water, reducing the spread of vector-borne diseases), support the sense of community, fight social exclusion and isolation (individual and community benefits, physically, psychologically, emotionally, and socio-economically), generate opportunities for urban–rural connection, increase the local attractiveness [13] and protect and promote cultural heritage (integrating and bringing to life local history, cultural values, and previous traditional use of the area) [14]. Reusing the underused and abandoned green areas improves the quality of the urban environment.

### 1.2. Landslides, the Shaping “Actors” of Urban Areas

Although natural disasters occur worldwide, their impact has been observed to be greater in developing countries. These events are mostly perceived as uncontrollable events in which a society undergoes severe danger, disrupting all or some of the essential functions of the society. Their occurrence is due to the two main factors: geographical location and geological/geomorphological settings of the given area. Among these factors, historical development represents an important factor which is mostly influenced by the changing economic, social, political and cultural conditions. These consequently act as factors creating high vulnerability to natural disasters, reflecting on economic, social, political and cultural vulnerability (e.g., [15,16]).

**Figure 1:**
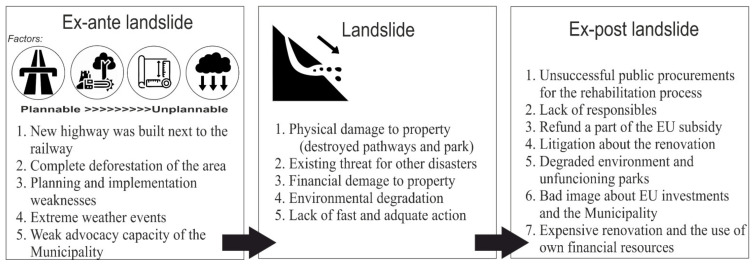
Framework of landslide process and outcomes used in this investigation.

On the other hand, natural hazards (sudden, disastrous events with high possibility to cause harm to various aspects of human life) like landslides involve publicly observed and monitored state-controlled interventions requiring special financial conditions. The redevelopment processes are determined by several factors, but information is considered one of the most important in the process [17]. During post-environmental catastrophe interventions (a complex interaction between nature and society, which enables long-term hazard management in accordance with the needs and capabilities of local communities) professionals usually create cost-benefit analyses based on the traditional utilitarian approaches and evaluate processes, despite the fact that such a situation should be dealt with using the most favorable intervention techniques for the local population [18,19]. Landslide-related disasters often involve urban development issues, which are considered rather as inherent to urbanization process, urban sprawl, and socio-economic changes, such as land disputes [20], or transformation of public spaces [21]. Nevertheless, these processes often play an integral part of the cause of a landslide and remediation process of the disaster-prone areas [22]. Disregarding the multiple dimensions of a landslide and its effects can be the root of the problem as well (Figure 1).

As pointed out by [23], slope instability is one the most widespread natural hazards; although it is not generally perceived as being as disastrous as other hazard types it can have a profound impact on populated areas. In-depth investigation of such events requires interdisciplinary approaches [24], which take into account not only geomorphological or geological factors, but also political, economic and social aspects. Natural hazards like landslides are aggravated by anthropogenic factors, such as: accelerated constructions, tree-cutting on steep slope areas, and obstruction of the drainage system [25]. A landslide area is a reasonable proxy for landslide magnitude and its destructiveness [26,27]. In this sense, landslide disasters are often the result of disorganized land-use changes, disregarded geomorphologic and geologic features, ignored and overridden regulations, and an uncontrolled urbanization process combined with extreme weather events [16,28,29], mainly flash floods and rainfall. Urban landslides represent a primary geologic hazard in many areas, especially coastal and riverside areas threatening public and private values, or properties [30,31]. In some cases, like hilly areas, rehabilitation requires additional planning and specific measures for dealing with natural disasters. Another common solution consists of non-structural measures (limitations in land use, town planning regulations, emergency plans) that allow activities to continue without slopes being stabilized [32]. For those areas where landslides are inactive, investments are usually permitted with the imposition of additional restrictive conditions. In this situation the measures are included in a major update of the development plan, resulting in the limitation of further development possibilities, important issue for the owners of these areas as the property value will most likely decline below the market conditions. The restrictions, reflecting the changes in land use, influence the increase or decrease in property value [33]. Therefore, disasters (as social construction) can affect investments or infrastructure at the county level. As a consequence, land management and planning of new activities require careful and reliable risk management measures [34,35].

The objective of this study is to analyze the relationship between selected meteorological parameters, their occurrence and temporal distribution in an environment susceptible to landslides due to the specific sedimentological and human land-use characteristics. In doing so, the authors identify and outline causal factors related to climate variability, geology, and human activity reflected via land use. This approach is found to be rather effective in neighboring countries (on local level), which are trying to implement sustainable approaches for effective mitigation of the hydro-meteorological factors influencing slope instability in urban environments. Therefore, results from this study could facilitate the promotion of adequate mitigation strategies and risk education, leading to vulnerability reduction and increased resilience respectively. The manuscript is organized as follows: 1. Introduction; 2. Study area; 3. Data collection and processing; 4. Results and discussion; 5. Management implications and mitigation measures, and 6. Concluding remarks.

## 2. Study Area

**Figure 2:**
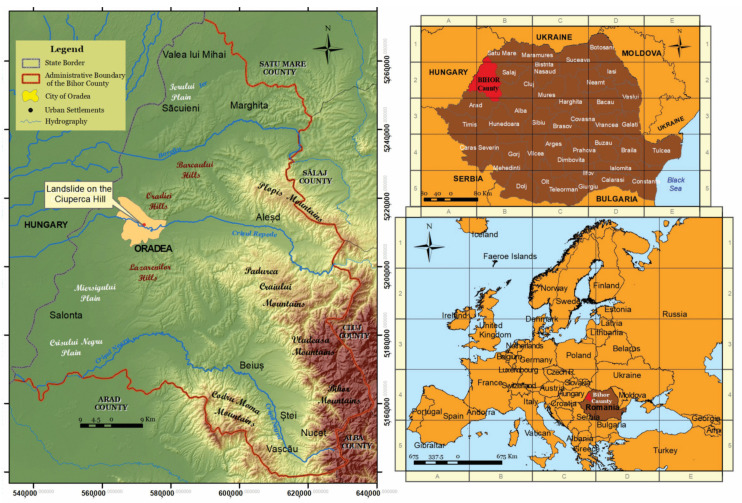
The Ciuperca Hill location within Bihor County, Romania. Data source: [38].

The city of Oradea (Figure 2) (pop. 221407) [36] is located in north-western Romania, close to the state border with Hungary, representing an important border crossing point towards central and western Europe. The development of Oradea over time was conditioned by the natural settings, as the settlement is located at the contact between two different geographical units (the plain and the mountain). Its location on the immediate edge of the mountain area (west of the Apuseni Mountains), as well as its positioning at the exit of the Vad-Borod depression along the banks of Crişul Repede River, influenced the spatial and functional development and enhanced the transportation function. The physical properties of the area managed to keep the settlement compact, and further imposed an uneven development on the two banks of the asymmetrical valley of the Crişul Repede River. From the initial medieval nucleus (fortress and fairgrounds), the settlement expanded to the flat areas accompanying the terraces of the river, with a wider extension on the left bank side, which is flatter and lower in altitude, allowing a larger spatial expansion and development. The city then climbed the neighboring hilly area (Oradiei Hills), which was in time integrated into the built-up area of the city [37].

**Figure 3:**
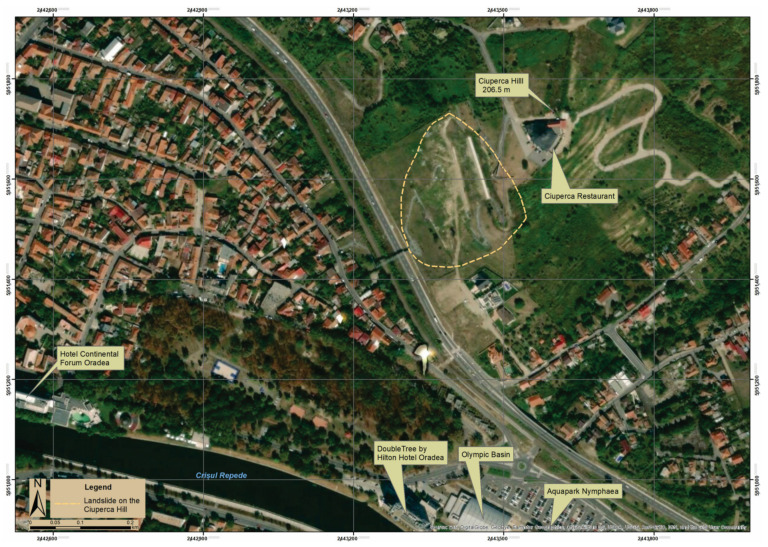
The Ciuperca Hill–Crișul Repede River Green Areas. Data source: modified after [40].

The study area is located in the city of Oradea and is known locally as Ciuperca Hill (elevation of 206.5 m) (Figure 3). It is the southwestern terminal part of the Oradiei Hills (component of the Western Hills of Romania). The hills are developed northeast of the city and look like long east–west oriented ridges. The neighboring plain area’s relief is displayed longitudinally, with higher altitudes (150–200 m) in the Pleistocene deposits of the eastern part and lower altitudes (80–120 m) in the Holocene deposits of the western part [39].

### 2.1. Geological Substrate

The Oradea surroundings are located on the upper Holocene lower level of a floodplain. The plain is constituted of gravel and sands. There are also five higher positioned fluvial terraces and glacial deposits located on the slopes of the Apuseni Mountains. Sands in alternation with sandy clays crop out from the terraces. The glacial deposits are constituted of gravel and sands of the Upper Pleistocene. The fluvial plain is characterized by a dense network of abandoned river beds, with E–W and SE–NW directions, which can be clearly recognized in remote sensed images (aerial photographs and satellite images), as well as in the field, and can be easily mapped [41].

**Figure 4:**
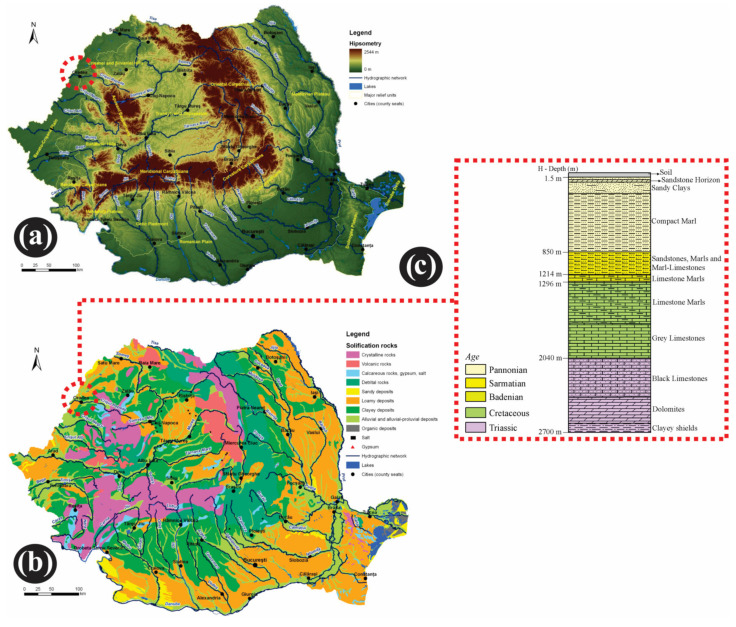
Major relief units (**a**), and solification rocks in Romania (**b**) with the Oradea area stratigraphic chart (**c**). Data source: modified after [46,47].

In a geological sense, the analyzed area is located at the contact of two main units: the Pannonian Basin (having the base dislocated into numerous fragments), and eastwards the orogen area represented by the Apuseni Mountains (part of Western Charpatians). The Crişurilor Plain, subunit of the Romanian Western Plain, was formed in the conditions of the continuous subsidence of the Pannonian Depression, and in the context of withdrawal and filling of the Panonnian Lake. The plain is composed of alternative Tertiary and Quaternary deposits up to 600 m depth (consisting of fine sand, clays, marls, gravel), overlapping the crystalline base [42,43]. The deposits of the Western Hills have considerable thickness and are composed of sedimentary formations (marls, tiles, clays, sands). Pannonian deposits (with total thicknesses > 1000 m, encompassing the Lower and Upper Pannonian), are those that occur to the topographical surface and they influence, by their physico-mechanical and chemical properties, most of the morphology and dynamics of the current geomorphological processes. The lower Pannonian deposits, occurring in the Ciuperca Hill sector are comprised of thick layer of compact marl. On the the other hand, the upper Pannonian sediments comprise a horizon of average thickness of ~3.5 m (Figure 4). On the plain-hill contact, the Quaternary formations of the plain are meeting the Neogene (Pliocene) formations of the hills [43,44,45].

### 2.2. Climate Properties

The climate of the investigated area is continental-temperate with oceanic influences in the central and western parts. The rainfall regime displays yearly, seasonal and monthly variability, directly influencing slope stability. The highest quantities of precipitation are recorded during the warm season, characterized by increased thermal convection (mainly in the plains and hilly regions) as well as during the years in which cyclonic and frontal activity prevails [48]. In the hilly regions, mean annual precipitation ranges between 400 and 600 mm (approximately 640 mm for the Oradea region). As pointed out in the study by Balteanu et al. [48], climate warming, reflected by a slightly increasing trend in mean annual temperature and a rise in torrential rainfall over the 1962–2000 period, alongside land cover modifications, may have favored a more frequent occurrence of flash floods and landslides [49]. According to Balteanu et al. [48] higher precipitation amounts are recorded in the south-western, western and north-western parts of the Romanian Carpathians, while a decreasing trend in mean annual precipitation has been recorded in the Southern and Southeastern Carpathians during the 1990–2005 intervals.

### 2.3. The Ciuperca Hill, between Culture and Sustainability

The Ciuperca (Mushroom) is an iconic part of recent and historical times in Oradea. The hill is a symbol of the city, as its elevation gives a perfect spot for a lookout over the settlement. At the same time, the area was used for multiple purposes throughout history [50]: agricultural (vineyards between 1782 and 1785, as the roots of the vines were an important factor for the stabilization of the slope), religious (during the Habsburg administration, the hill was named Kálvária Hill (Calvary) and it hosted on its top a Roman Catholic Neoclassical chapel), and recreational (the Communist regime demolished the chapel in 1955 [13] and built in its place a restaurant with a round mushroom shape, after which the hill was named: the Ciuperca (Mushroom) Hill).

For several decades this was one of the main attraction sites of the city because of its beautiful perspective over the settlement and the Crişul Repede valley, but after 2000 the hill’s recreational use was abandoned. For many years the site was neglected, the park area was underused, the tourist potential was not exploited, Ciuperca as a facility was not sufficiently attractive, and the view was not integrated in the local touristic offer. Even so, Ciuperca Hill represented one of the most important natural green areas of the city of Oradea, being a fragile environment, vulnerable and very sensitive to urban expansion. The local urban regulatory framework supports the rehabilitation and regeneration of the abandoned and degraded green areas, in an effort to increase the surface of the green areas in the city of Oradea [51].

The local strategical documents [52,53], together with scientific research [54] explain that the favorable exposure of the northern slopes of the city is attractive for development (residential, commercial, recreational), but the changes in land use (the reduction of traditional vineyard and orchard surfaces) and new development (constructions) reduced the geological stability of the slopes and increased the landslide risks. In addition, the site is affected by historical landslides, for example Zoltán Ozorai [55] reported a landslide in 1941 that lasted four days and affected a surface of 30 hectares.

In this context the rehabilitation of the abandoned and under-used green area of the Ciuperca Hill became inevitable, therefore it was made a priority of the project titled *Public garden on Ciuperca Hill-Development of natural habitats of protected tree species and spontaneous flora* (an investment worth 1,722,253 Euro for Oradea, part of the joint project of the Oradea City Hall and the local government of Körösszegapáti, funded by the Hungary-Romania Cross-Border Co-Operation Program 2007–2013) [56]). The project aimed to turn the area into an attractive space for visitors, and to develop the visiting facilities, protecting at the same time local natural and cultural heritage, and promoting an overall healthier environment. The initial 18,000 m^2^ park from Ciuperca Hill was expanded to 38,759 m^2^, after the Greek Catholic Church, which owned most of the land, transferred it to the Oradea City Hall. The site was a degraded green area, occupied by uncontrolled vegetation of trees and shrubs, with visiting trails and platforms ruined because of lack of maintenance and land instability [57].

**Figure 5:**
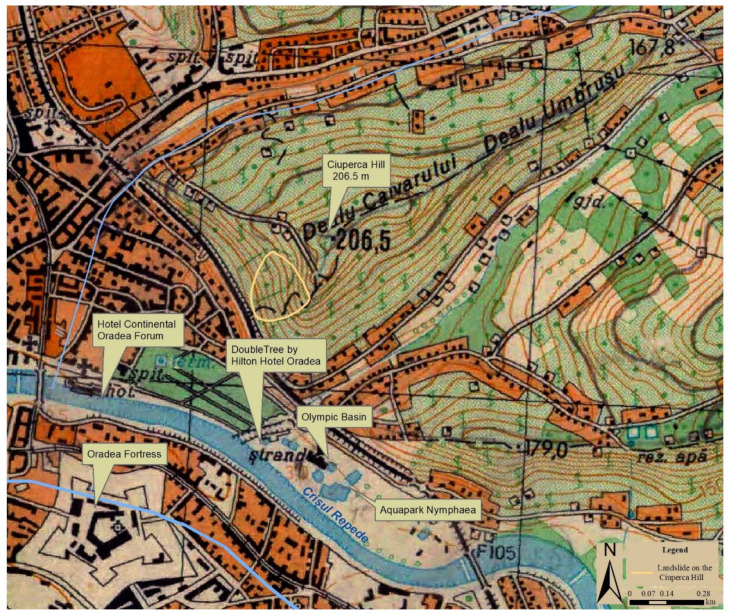
The complementarity of attractions, from the Ciuperca Hill–Oradea Fortress area. Data source: modified after [60].

According to the local authorities [58] the works on the public garden on Ciuperca Hill consisted of the planning of the area at the base of the slope for offering the access road from Olteniei street, construction of five panoramic points, a restaurant (offering a great view of the city) and a tourist information point. In addition, recreational areas, alleys, an open-air amphitheater (capacity 130 persons, for educational and cultural activities) and a pedestrian bridge over the railway and express road were part of the investment as well. For the stabilization of the land, works were carried out on the slope surface, consisting of support walls from natural stone, in close correlation with the construction elements mentioned above. The paths (considering the land topography, as higher slopes are avoided) take visitors through various attraction points, connecting the base of the slope with the top of the hill (where the panorama point is located). The Public Garden Ciuperca Hill was planted with trees, shrubs, perennials and spontaneous flora, and with vineyards [58]. The park can be reached from bottom of the slope, from the Brătianu park only by foot, across the pedestrian bridge over the railway and via the express road, built in the meantime. The new express road was constructed in the base of the hill as well, which added extra land-use challenges to the area, in addition to the deforestation process. The integrative urban planning was considered [59] as its redevelopment was complementing the neighboring attractions like Nymphaea Wellness Thermal Complex (located in the proximity, along the Crișul Repede River), or the recently rehabilitated Oradea Fortress (located on the opposite side of the river) (Figure 5). All of this was part of a complex transformation process, aimed at increasing urban attractiveness.

### 2.4. The Landslide of the Ciuperca Hill in Oradea

As pointed out, Romania is one of the European countries most affected by landslides, that have a major impact on human activity [48,49,61]. Mountains, hills and tablelands, which cover two-thirds of the country’s area, are particularly susceptible to landsliding processes. This is specifically pronounced when it comes to the hills and tablelands. Contemporary landslide studies in Romania consider many causal factors which could be used for risk assessment and management of landslide hazard [48,49,61,62,63,64,65]. Due to the susceptibility related to geological settings the most prominent ones are associated with climate properties and the presence of extreme weather events during the given period.

**Figure 6:**
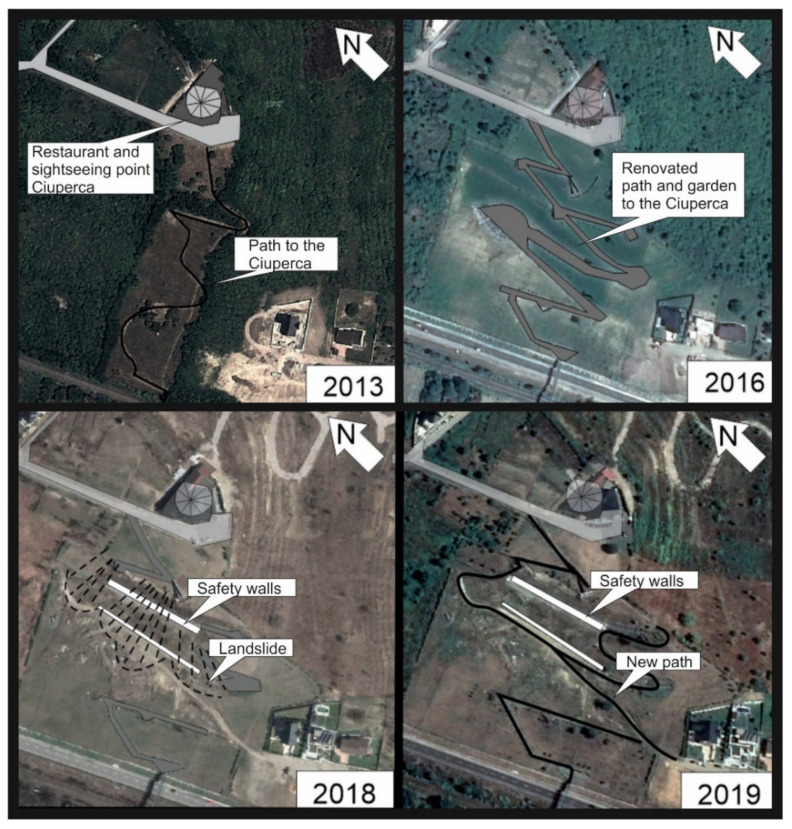
The evolution of the Ciuperca Hill landslide. Data source: modified after [40].

Between 12 and 13 of November 2016, the landslide area encompassed a surface of approximately 5300 m^2^ on the recently rehabilitated Ciuperca Hill in Oradea (Figure 5 and Figure 6). The 70 m landslide damaged the stone walls, the stairs, the promenade and paths, as well as water and electricity utilities and systems. The park became unusable, so the municipality of Oradea closed the hillside because of possible hazards (Figure 6). A specialist investigation was requested to determine the causes of the landslide. At the moment the landslide occurred (in November 2016), Ciuperca Hill had already been closed since the end of October 2016, as the Oradea City Hall urged the contractor to repair the works, after some parts of the trail split and cracked due to the unevenness of the soil. The repair costs were to be borne by the contractor, but the works were not undertaken before the landslide happened.

## 3. Data Collection and Processing

The presented study encompasses an interdisciplinary methodology for the holistic understanding of the interrelationship between atmospheric conditions and landslide occurrence, on one side, and humans and landscape on the other. Analytical tools, which include a system of variables interacting with one another, included historical cadastral maps and sources, geological/hydrogeological maps, contemporary remote-sensing observations, geo-technical documents and meteorological data.

### 3.1. Cartographic Material

Historical maps (Topographical Map of Oradea, scale 1:50,000, 1970) and documentation were used to demonstrate the landscape susceptibility to landslides and prior occurrences. A geological map for the surrounding area (Oradea sheet, Geological map of Romania, scale 1:200,000) was used for the aggregation of the lithostratigraphic data. These geological maps were produced by the Romanian Institute of Geology [47]. The Romanian map of solification deposits (scale 1:1,000,000) [66], and the relief units map of Romania was used as well [67].

### 3.2. Remote-Sensing Observations

Time series surface deformation was assessed using remote sensing techniques. In this research, Google Earth Satellite Recordings (GESR) were used. Satellite recordings were downloaded from an open-source server [38,40]. With the help of geographic information system (GIS) open-source software Quantum Geographical Information System—QGIS 3.16.5 (QGIS Development Team, Gossau, Zürich, Switzerland) and a System for Automated Geoscientific Analyses—SAGA digital elevation model (DEM) (Departments for Physical Geography, Hamburg and Göttingen, Germany), data were analyzed. The accuracy (resolution) of satellite data was 30 m of resolution (Table 1). These data were useful for very precise terrain analysis and classification of landscape and changes within the landscape itself [68]. The average error of downloaded data is 1%.

### 3.3. Meteorological Variables

Daily values of temperature and precipitation were obtained for the Oradea meteorological station (47°03′ lat. N, 21°90′ long. E and elevation of 140 m.a.s.l.) from the National Meteorological Service of Romania for the 2003–2017 period. Meteorological observations are in accordance with World Meteorological Organization (WMO) standards. Daily values of precipitation and air temperature were used to aggregate monthly and annual values.

Although there are many factors that influence the occurrence of landslides besides the precipitation factor (e.g., prevailing climatic conditions, soil type, vegetation cover, slope, terrain ruggedness etc.), the authors of this study focused on selected pluvial and drought parameters in order to fully describe the variability and occurrence of the main agent of wet mass movement events. Selected meteorological parameters were used in order to calculate rainfall erosivity indices such as precipitation concentration index (*PCI*), modified Fournier index (*MFI*), rainfall erosivity (*RE*) and erosivity density (*ED*), as well as factors affecting them at local scales [62] in the context of climate variability as an important prerequisite of soil erosion prevention and soil loss risk assessment [16,28,69] over the 2003–2017 period. The *PCI* characterizes rainfall variability through space and time, and has previously been used to identify potential patterns of climatic interaction with geological substrate that may trigger landslide events (e.g., [28]). It is also the basis for calculating the rainfall erosivity index, the *MFI*, which has been used to analyze rainfall aggressiveness and its correlation with other climatic variables contributing to catastrophic erosion (e.g., [16,17,28,69]). Rainfall erosivity indices were used because they represent not only the higher percentages of the annual total precipitation in a few very rainy days, but also the time and degree of concentration of the yearly total precipitation within a year. They have high potential to indicate and characterize wet mass movement events [70]. Because of the lack of long-term time series of rainfall data with high temporal resolution that can be used to calculate *USLE* or *RUSLE* rainfall erosivity, daily rainfall data have been used to estimate rainfall erosivity (*RE*). The erosivity density (*ED*) is highly reliant on rainfall intensity and influences event sediment concentration (i.e., soil loss per unit quantity of water). As a pluvial parameter (expressed as MJ *ha^−1^h^−1^), it shows the ratio of rainfall erosivity to precipitation and measures the erosivity per rainfall unit (in mm). The calculation of *PCI* and *MFI* have been performed following the approach of Oliver [71] and Arnoldus [72], while the *RE* and *ED* were calculated by following the approach of Li and Ye [73]. On the other hand, the drought index, such as *SPI,* is a multi-scalar in time and space, and it is a rather good parameter for comparing indices between different locations, and the frequencies of comparable extreme events [74,75]. This statistical indicator provides a more objective determination of drought phenomena when considering precipitation variability. Therefore, the *SPI*, as a meteorological drought indicator was included in this study since it is based on wide use and regional relevance. The *SPI* was calculated and classified according to the approach provided by McKee et al. [76], Bordi et al. [77] and Lloyd-Hughes and Saunders [78]. In this way, analysis of the aforementioned meteorological variables represents an important task when concerning the implications of erosion intensity in the investigated area. *R* statistical analysis software (*R* Core Team, The *R* Foundation for Statistical Computing, Vienna, Austria) was used for the calculation of the indices and data visualization.

### 3.4. Technical Documentation

Geo-technical documents were used to examine the interplay between geological settings and the landscape development plans, while the meteorological data were used to quantify the hydro-meteorological soil degradation indices. In this way, the authors were able to compare the results obtained and documented field registrations of the landslide events for the case study area with quantified erosion parameters. For this purpose, Technical Expertise for the Ciuperca Hill Public Garden landslide, the city of Oradea from 2017 was used [79]. This document identifies the causes of landslides and the appropriate rehabilitation measures, which can be used to describe the nature of dynamic erosional processes and landslide occurrence related to the interplay between landscape dynamics and climate characteristics of the investigated area, associated with anthropogenic influence respectively.

**Figure 7:**
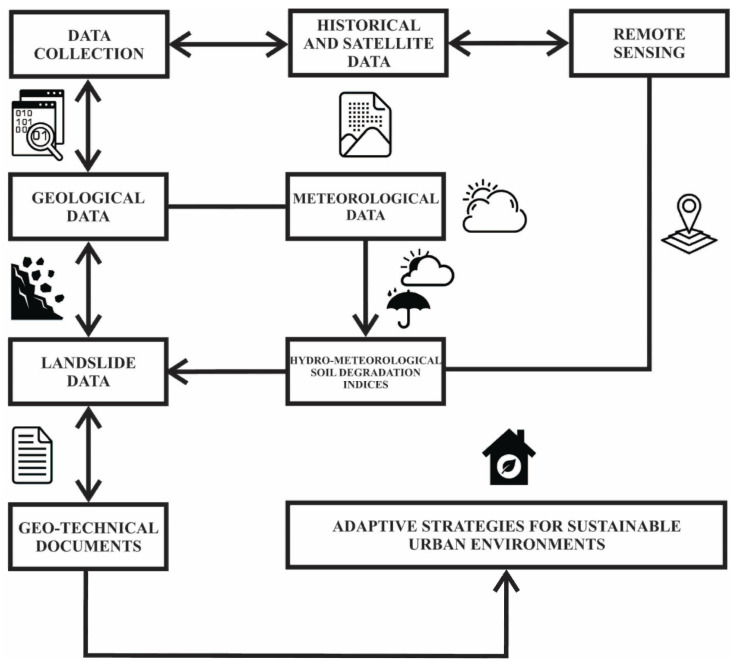
Flow chart of the procedures used in this research.

All the procedures and approaches used for the purpose of this research are presented in the following flow chart (Figure 7).

## 4. Results and Discussion

**Figure 8:**
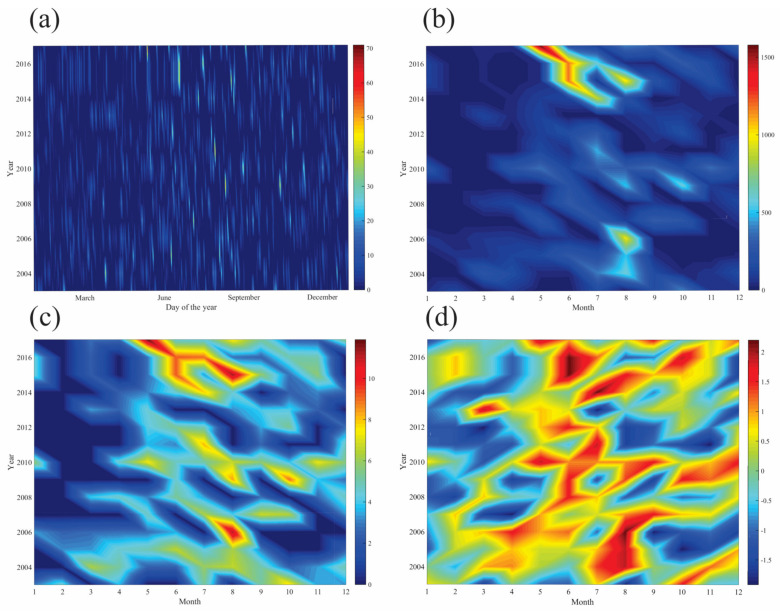
Temporal variability of selected meteorological and erosion parameters: daily precipitation amount in mm (**a**); rainfall erosivity—*RE* in MJ*mm*ha^−1^h^−1^ (**b**); erosion density—*ED* in MJ *ha^−1^h^−1^ (**c**) and standardized precipitation index—*SPI* (**d**). Data source: calculations based on [80].

Obtained results of the *PCI* suggest the presence of uniform, moderate and irregular precipitation concentration for the observed period (values from 9.2–15.1). The *MFI* based on monthly mean values yields diversity in terms of erosion classes: 13.3% of very low, 33.3% of low and 33.3% of moderate aggressiveness, with several extreme values (144, 133 and 132 respectively), indicating very high erosivity classes for 2010, 2015 and 2016. Analysis of daily precipitation displays the occurrence and intense distribution between May and September. Higher *RE* is observed for 2014–2017, occurring between April and August. *ED* follows the pattern of *RE* and indicates high-intensity events from April until October. *SPI* index reveals the greater presence of various wet classes, especially during the month of June in 2015 and 2016, where index values of 2.2 indicate the occurrence of extremely wet conditions. The majority of drought index classes vary from moderately to extremely wet (index values of 1.2–2.2 in the period June–October) (Figure 8a–d).

**Figure 9:**
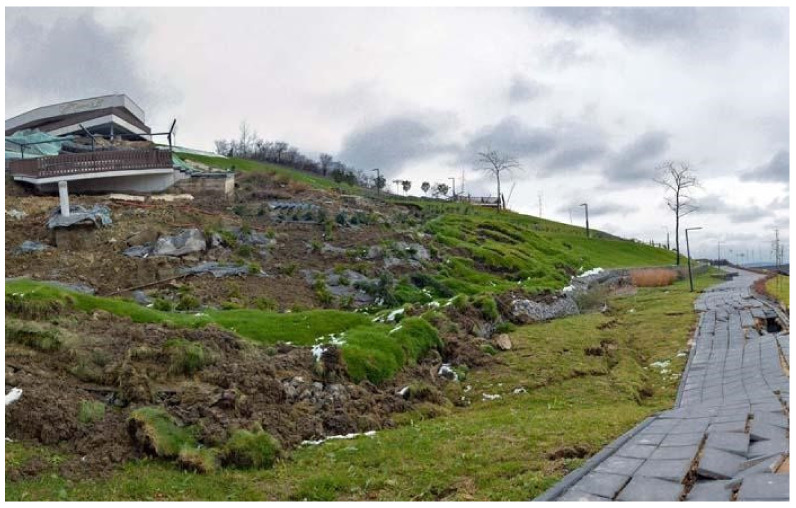
Perspectives over the landslide from Ciuperca Hill in Oradea (12–13 November 2016). Source: [81].

**Figure 10:**
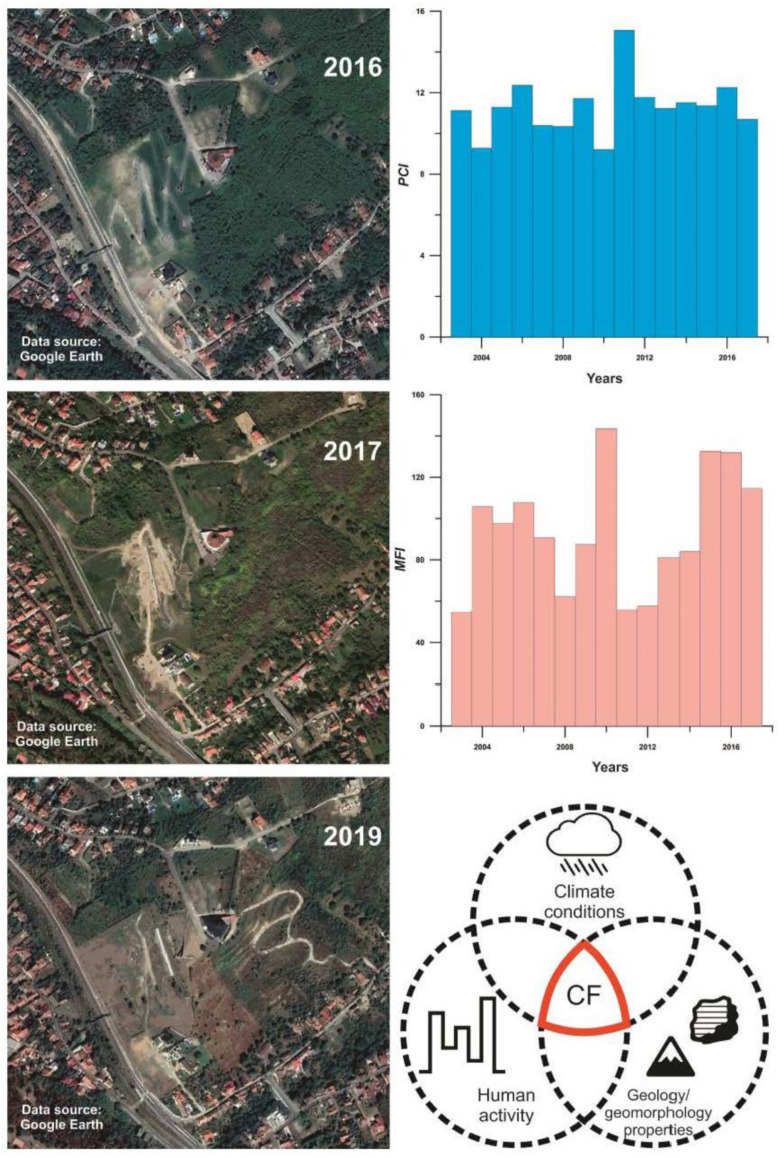
The time sequence of landslide event. Data source: derived after [40,80].

The precipitation values for 2016 indicate higher precipitation rates just prior to the largest landslide event. The corresponding *MFI* values for 2015 and 2016 (133 and 132 index units respectively) fall within a very high erosivity class. These suggest that the high precipitation recorded in 2016 occurred in a few torrential events over a short period of time, thus increasing the susceptibility of the geological substrate to erosion and landslides. It is important to note that activation of the landslide event in 2016 was additionally backed up by mishandled activities preformed on the hill (Figure 9). Results from this study correspond well with the findings of Bălteanu et al. [49], who produced a national-scale landslide susceptibility map of Romania in a European methodological framework. According to this research, the Oradea area has low values when it comes to distribution of densities of landslides at the municipality level (0–10 landslides per 100 km^2^). In regard to spatial analysis units, 0.7% of the area has high values of slide and flow susceptibility, which are outlined and addressed in the presented study (Figure 10).

As previously pointed out, the geological substrate predominantly comprises sandy clays on more compact marl with a high degree of plasticity and hence highly susceptible to erosion and landsliding when climatological conditions are suitable. Bălteanu et al. [48] show that the interaction between land use/land cover and landslides is a very complex phenomenon, with the frequency and distribution of landslides being significantly related to the changes in these factors. Therefore, accelerated urbanization and reduced vegetation cover has intensified slope instability in the study area [37]. Variations of volume and moisture content in given Pannonian sediments, accompanied by intense humid periods (spring, autumn), accelerate terrain instability as well. This process is presented by piping-plastic movements, where the landslide takes place on a clay slip bed, most of it being covered by shrub vegetation [82]. Hence, the causal factors (*CF*) [62] of this event can be found in joint climatological characteristics of the investigated area, properties of the geological substrate, and human activity that further contributed to the intensive change of landscape (transformation of tree and grassland into tillable land cover) and acceleration of slope instability (Figure 10). These findings correspond to the field observations of the 2017 Technical Expertise for the “Ciuperca Hill” Public Garden landslide from Oradea [79], requested by the Oradea City Hall, in order to identify the causes of landslides and rehabilitation measures. In addition, they generally correspond well with similar studies conducted in neighboring countries (e.g., [16,69]).

## 5. Management Implications and Mitigation Measures

**Figure 11:**
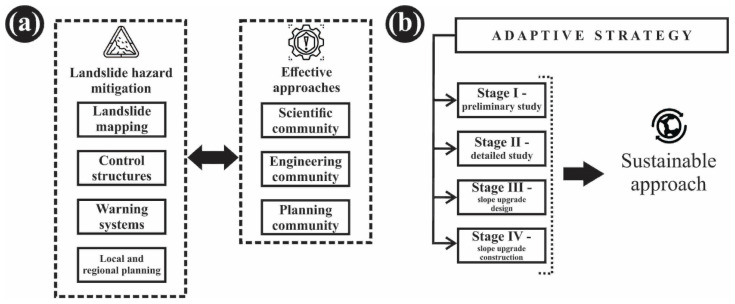
The landslide hazard mitigation approach (**a**) and sustainable adaptive strategies (**b**). Data source: modified after [83,84].

According to Gori et al. [83], qualitative landslide hazard mitigation generally involves landslide mapping, control structures, warning systems, local and regional planning. Most effective approaches include a combination of the aforementioned strategies, with good coordination between the scientific, engineering and planning communities (Figure 11a). Furthermore, Larsen [84] highlights that communities can reduce their exposure to landslide hazard if they understand the threat, its potential impact, and mitigation options. National policy in disaster risk reduction field in Romania is expressed through various legislative documents for risk types, administrative authorities, public institutions, and specialized institutions with responsibilities in disaster prevention and response management. Therefore, the most adequate approaches need to be carried out in order to enhance and implement an institutional and legal framework fully harmonized with European Union (EU) requirements, following examples of good practice which stand in good agreement with sustainable principles [85]. In this way, local community can address and solve problems related to risk management in a quality manner. Even though landslides along the Ciuperca Hill slopes present a lasting problem for the inhabitants of Oradea, landslide control, mitigation, and management are not administered in a sustainable manner. Local officials only take action after landslide occurrences, resulting in adverse economic impacts for the area. A potential lasting solution for the present situation lays in preparedness, requiring Oradea officials to introduce and conduct qualitative mitigation strategies by implementing a long-term slope stabilization program. Examples of good practice are evident in man-made slopes worldwide (e.g., [16,69,86,87,88]). This adaptive strategy would imply a four-stage program (instead of a proposed three-stage program) for the design and construction of slope upgrading as presented on Figure 11b. Current rehabilitation of the abandoned and under-used green area of Ciuperca Hill, a cultural symbol of Oradea city, from the standpoint of sustainability and interplay between natural and anthropogenic processes that occur in susceptible natural environments, is based on the three main approaches of the proposed adaptive strategy. This adaptive strategy takes into account the perspective of local authorities and 2017 official technical expertise [79] and implies a three-stage program for the slope upgrading and stabilization:

1. Strengthening restaurant and tourist information point buildings. This involves two categories of work:
  - Groundwater drainage—three drains (depth ditches with a width of approximately one meter) are to be constructed along the hill, so that the bottom of the drains is at the boundary between the stable land and the land susceptible to sliding;
  - Surface drainage—drains are required on the support walls and on the access roads area that collect the water and transport it downstream for discharge into the emissary.
2. Stabilization of the slope—at the bottom of the area, drilling of pilots will be accomplished, constituting a support area.
3. Rehabilitation works: architectural (architectural works on the restaurant, landscape observation point, alleys, visiting paths, amphitheater etc.), structural (for stabilizing, structurally constructed), landscaping (planning with tree planting, shrubs etc.) occur in 2019–2020 within the framework of the project “Redevelopment of the Public Garden Ciuperca Hill (2,600,000 RON)” [89,90].

Beside these stages, a slope information system is needed to create public awareness concerning potential hazards related to wet mass movements, and to cultivate a responsibility towards maintaining slope safety within own property boundaries. A future approach should include landslide susceptibility mapping by using the Landslide Susceptibility Mapping Tool Pack (LSM Tool Pack, Department of Civil Engineering, Bolu Abant Izzet Baysal University, Bolu, Turkey) as proposed by Sahin et al. [91]. Public education regarding slope maintenance and landslide reporting is needed as well. Slope stabilization requires substantial engineering works based on the principles of removal, reinforcement, retention, and replacement. Results presented in this study may contribute to improved understanding of the local dynamics of the main climatological agent of pluvial erosion in terrains susceptible to landsliding. Furthermore, this could aid in creating suitable mitigation strategies to avoid or reduce the impacts of landslide occurrence not only in the investigated area, but in the surrounding regions (with similar geological strata) as well.

## 6. Concluding Remarks

Rehabilitation of the abandoned and under-used green area of Ciuperca Hill, a cultural symbol of Oradea city, was thoroughly addressed in this paper from the standpoint of sustainability and interplay between natural and anthropogenic processes that occurs in the given case study. Urban, cultural and environmental values have been put into danger by the complex of factors that caused the landslide. The slope stability, being a very complex phenomenon, requires a multidisciplinary approach to survey and mitigation strategies. The approach to solve problems associated with landslides is one of the most challenging, but it is also the most suitable issue for a good implementation of spatial planning strategies along with valid land use. A detailed analysis of climatic and geological/geomorphological factors is of great importance when identifying the causal factors of landslide events. To reduce future infrastructure damage and prevent the development of landslide events in the Oradea area, it is necessary to introduce and conduct qualitative mitigation strategies by implementing a long-term slope reinforcement program. A successful strategy is dependent on a combined scientific and engineering approach and management of environmental resources at both local and national levels. In this way, urban environments that are highly susceptible to hydro-meteorological factors can achieve a satisfying level of sustainability. The results presented in this case study can, furthermore, contribute to creating suitable mitigation strategies in order to avoid or reduce the impacts of landslide hazard occurrence not only in the investigated area, but in the surrounding regions (with similar geological strata) as well.

## Figures and Tables

**Table 1:** Satellite recordings properties used in this research.

Satellite	Cover Type	N	Accuracy	Aster DEM
Google Earth				
Landsat 8	General	2.333	0.95	0.1
USGS				
Landsat 8	General	2.756	0.97	0.05

N-number of attempts; Accuracy, DEM—digital elevation model. Data source: modified after [38,40].

## Data Availability

Part of the data was collected by the authors during their own field research, while another part was kindly offered by the National Meteorological Administration, Oradea Meteorological Station (Romania).
